# Clinical and Experimental Immunomodulation 2016

**DOI:** 10.1155/2017/3152956

**Published:** 2017-01-16

**Authors:** Lenin Pavón, Hugo Besedosky, Oscar Bottasso, Marco A. Velasco-Velázquez, Moisés E. Bauer

**Affiliations:** ^1^Department of Psychoimmunology, National Institute of Psychiatry “Ramón de la Fuente”, Calzada México-Xochimilco 101, Colonia San Lorenzo Huipulco, Tlalpan, 14370 Mexico City, Mexico; ^2^Research Group Immunophysiology, Institute of Physiology and Pathophysiology, Philipps University, 35037 Marburg, Germany; ^3^Instituto de Inmunología Clínica y Experimental, CONICET-Universidad Nacional de Rosario, Suipacha 590 CP, 2000 Rosario, Argentina; ^4^School of Medicine, National Autonomous University of Mexico, Avenida Universidad 3000, Coyoacan, 04510 Mexico City, Mexico; ^5^Institute of Biomedical Research, Pontifical Catholic University of Rio Grande do Sul (PUCRS), Avenida Ipiranga, No. 6690, 90610-900 Porto Alegre, RS, Brazil

Inflammatory response (IR), which is crucial in injuries or infected anatomical regions, also generates systemic effects, regulating multiple physiological processes. Those effects depend on the concentration of soluble mediators like cytokines, chemokines, and other inflammatory molecules. For example, concentrations of soluble mediators around 10 nM are enough to induce a neuroendocrine response. The diverse systemic effects triggered by IR are plastic and continuously modified by fluctuations of circulatory levels of hormones, neurotransmitters, and mediators of inflammation. These feedback loops are possible by the constitutive expression of receptors for hormones, neurotransmitters, and cytokines on leukocytes, which modulate key cellular functions like proliferation, differentiation, and the secretion profile.

That is the reason whereby the constant research on clinical and experimental parameters that modulate is of great importance. This third special issue on clinical and experimental immunomodulation compiles a selection of high quality works on the field.

For the clinical immunomodulation research, we present the work of M. Żabińska et al., who analyzed quantitative changes of CD3+CD8+CD28-T cells and Foxp3 expression in patients with lupus nephritis. Flow cytometry data revealed increased CD3+CD8+CD28-T cells and a lack of Foxp3 expression on such cells, pointing out to a favoring role of these lymphocytes in the increased inflammation seen in active lupus patients. In a paper that focuses on the treatment of diseases in which TNF-*α* is pathological, M. P. Miranda-Hernández et al. compared the physicochemical and biological properties of a biosimilar etanercept along with its performance in rheumatoid arthritis (RA) patients. Their results showed a full correspondence of the primary and higher order structures between the biosimilar and the reference product. Despite being highly heterogeneous, both compounds exhibited no significant differences in the in vitro inhibition of apoptosis nor in the pharmacodynamic pattern seen in RA patients. I. Siloşi et al. analyzed the serum concentrations of IL-13 and IL-17 in patients with early RA as well as the link of these cytokines with disease activity scores and levels of some autoantibodies. They found that high concentrations IL-13 and IL-17 correlate with disease activity. In the case of the groups with severe and moderate RA, the IL-13 concentrations were statistically higher than in the group of mild RA. Also, IL-17 concentrations increased proportionally with the disease activity. According to these findings, both cytokines have a great potential like RA biomarkers as well as for diagnostic applications.

W.-P. Lee et al. demonstrated that 1,25-dihydroxyvitamin D3 treatment in vitro of monocyte-derived DC results in a semimature phenotype and anti-inflammatory cytokine profile as compared to conventional DC in both healthy controls and MS patients. Cryopreservation of DCs did not affect this profile. A. Gutiérrez-Hoya et al. demonstrate that G-CSF treatment in vivo induced pro- and anti-inflammatory T-cell profiles in healthy controls and in patients with graft-versus-host disease. In particular, they reported expansions of Tc1, Th1, Tc17, and CD8+IL-10+ T cells following treatment. S. Harm et al. tested different commercially available adsorbents for the effective removal of cytokines from plasma. This work has an important clinical significance in severe inflammation, sepsis, and septic shock, among other diseases with significant inflammatory component. Their report confirms the hypothesis that cytokine reduction in blood is required in order to reduce endothelial cell activation.

Finally, we must mention the interesting work by D. Ramírez-Ramírez et al., which shows that the production of specialized NK lymphoid lineages can be strengthened by dialyzable leukocyte extracts (DLE) from their early steps of differentiation. The study sheds some light on lymphopoiesis regulation by self-components and suggests that DLE may promote innate NK cell reconstitution during emergency conditions such as infection or malignant diseases.

In this issue we also present several works on experimental immunomodulation. The work by L. L. W. Ishikawa et al. demonstrated that pretreatment with several doses of proteoglycan (PG) partly protects BALB/c mice from the experimental arthritis induced by PG plus dimethyl-dioctadecyl ammonium bromide. Protection coincided with a lower production of IFN-*γ* and IL-17 along with an increased release of IL-5 and IL-10 by PG-stimulated spleen cells. L. Zhang and C.-Q. Xia showed that transfer of UVB-treated immature DC from BALB/c induces tolerance in C3H mice. The underlying mechanisms were related to PD-1/PD-L1 interactions in tolerant mice and essential for controlling alloantigen-specific T cells. W. G. Bain et al. showed that macrophages from wild-type mice exposed to cigarette smoke for 5 weeks, followed by intratracheal instillation of* Pseudomonas aeruginosa* and 35–40% oxygen, exhibit improved survival and reduced lung CFUs and inflammation. Macrophages from these mice expressed less TNF-*α* and more scavenger receptors. This finding suggests that the protective role of low-dose oxygen may enhance macrophage phagocytosis.

In view of the influence of the major histocompatibility complex on the course of HBV infection, L. Wang et al. reviewed evidence that, in general terms, suggests that single nucleotide polymorphisms near certain HLA gene loci are linked to different infection outcomes as the spontaneous viral clearance or its persistence resulting in liver cirrhosis and hepatocellular carcinoma in chronic carriers as well as the efficacy of anti-HBV treatment and their specific vaccination. Since M1 and M2 macrophages may display antitumoral or protumoral activities, respectively, K. Chimal-Ramírez et al. performed a study wherein cell line-derived or primary monocytes were subjected to M1/M2 polarization procedures and to conditions for skewing monocytes to a protumoral function. Except for the fact that IDO enzyme and CD86 M1 markers correlated with M1 polarization, protumor differentiation was not associated with a clear separation into M1 or M2 phenotypes. S. Cai et al. showed that low-dose* Lactobacillus rhamnosus* GG stimulates DC to induce greater Th1 polarization in T cells. This suggests an important immunomodulatory effect with a potentially relevant clinical effect, especially in antitumor therapy.

The study by R. Flores-Fernandez et al. demonstrated that PRL promotes self-reactivity by analyzing the effect of PRL in B-cell tolerance models employing WEHI-231 cells or immature B cells. PRL rescued WEHI-231 cells from cell death by decreasing the apoptosis induced by the cross-linking of the B-cell antigen receptor; a similar effect was found in immature B cells from lupus prone MRL/lpr mice. Y. Wang et al. analyzed the frequency and function of circulating T follicular helper (Tfh) cells in patients with psoriasis vulgaris as well as their presence in skin lesions. Results showed both increased frequency and activation of Tfh cells, correlating with disease severity as well as an increased presence of Tfh cells in affected skin. Given the role of lipopolysaccharide (LPS) in neurodegenerative diseases, Liu et al. carried out a study assessing the protective effects of epigallocatechin gallate (EGCG), the major component in green tea, on LPS-mediated inflammation and neurotoxicity. EGCG inhibited the LPS-mediated production of inflammatory cytokines by immune cells as well as the synthesis of reactive oxygen species in LPS-exposed neurons, highlighting the potential neuroprotective role of EGCG.

L. Paskova et al. compared the effects of a potential immunomodulator, natural polyphenol N-feruloylserotonin (N-f-5HT), with methotrexate (MTX), in the rat model of adjuvant-induced arthritis (AA). Both compounds reduced inflammation-associated surrogates and the transcription levels of TNF-*α* and iNOS in liver. Unlike MTX, N-f-5HT lowered IL-1*β* plasma levels and its mRNA expression in the liver and spleen of AA rats, implying a potential benefit of combined treatment with N-f-5HT and MTX in joint inflammation. M. Yuan et al. showed that CXCL1 serum levels arise in a lung cancer model induced by 3LL cell inoculation. Changes in CXCL1 contributed to tumor-associated neutrophils inhibition. In turn, this affects CD4+ and CD8+ T cells activation, allowing tumor growth. C. Chi et al. report that the compound “kaempferol 3-a-L-(4-O-acetyl) rhamnopyranoside-7-a-L-rhamnopyranoside (SA)” isolated from* D. crassirhizoma* has the capacity to stimulate the head kidney macrophages from the fish* Ctenopharyngodon idella*. This natural immunostimulant could be a potential substitute for antibiotics and chemicals in aquaculture practices.

In this issue we also present eight interesting reviews. J. S. Apostólico et al. compile, in a very interesting review, knowledge of the adjuvants commonly used in experimental and clinical settings with emphasis on their mechanisms of action. They also highlight the requirements for licensing new vaccine formulations. S. Xia et al. present a review about “inflamm-aging” in which they discuss studies of inflammation in old patients. The authors explore the concept of inflamm-aging with its pathological features and mechanisms. They also suggest therapeutic strategies that could be useful in Alzheimer's disease, atherosclerosis, heart disease, type II diabetes, or cancer. W. Wan et al. review the roles of HMGB1 in neuropathic pain. HMGB1 is an alarmin released by damaged tissues as well as by leukocytes. It may induce proinflammatory actions through binding in Toll-like receptors (TLRs) and RAGE and NMDA receptors; hence, it is considered as a therapeutic target for neuroinflammation.

R. Arreola et al. present an interesting review about dopamine (DA) features to modulate the immune response and generate changes in cellular phenotype and function. Leukocytes have the molecular machinery to synthesize and respond to this catecholamine but the expression of this machinery is dependent on the leukocyte type, the state of cellular activation, and the concentration and exposure time to DA. Some diseases present alterations in dopaminergic transmission in CNS and peripheral nervous system affecting the immune system modulation and causing the complications of the disease. In the review by E. A. Ivanova and A. N. Orekhov, the implications of abnormal monocyte activation and the acquisition of pro- or anti-inflammatory macrophage activities in disease states, like atherosclerosis or neoplasms, are discussed. Development of monocyte/macrophage activation tests may also be valuable in diagnostic or prognostic terms. I. L. Vladimirovna et al. review the properties and mechanisms whereby two distinct populations of immature cells, mesenchymal stem cells and myeloid derived suppressor cells, mediate immune regulation. The authors discuss cell similarities, discrepancies, and potential clinical applications.

Y. Zhou et al. contribute with an interesting meta-analysis about tolerogenic DCs and immunosuppressive (IS) therapies in multiple models of transplantation. They conclude that Toll-DC therapy significantly prolonged multiple allograft survival and further prolonged survival with IS. G. Hurtado-Alvarado et al. present a review about blood-brain barrier disruption observed in sleep-deprived rodents. The disruption could be associated with the increase of inflammatory mediators induced by sleep loss that are able to modify the expression of tight junction proteins in the brain microvasculature. The effect of these inflammatory molecules should be taken into account for the study of general consequences of sleep loss including the risk of developing neurological and neurodegenerative diseases.

